# From waste to protein: a new strategy of converting composted distilled grain wastes into animal feed

**DOI:** 10.3389/fmicb.2024.1405564

**Published:** 2024-05-31

**Authors:** Lei Yu, Zichao An, Dengdeng Xie, Diao Yin, Guopai Xie, Xuezhi Gao, Yazhong Xiao, Juanjuan Liu, Zemin Fang

**Affiliations:** ^1^School of Life Sciences, Anhui University, Hefei, China; ^2^Anhui Key Laboratory of Modern Biomanufacturing, Hefei, China; ^3^Anhui Provincial Engineering Technology Research Center of Microorganisms and Biocatalysis, Hefei, China; ^4^Anhui Golden Seed Winery Co., Ltd., Fuyang, China

**Keywords:** distilled grain waste, composting, microbial inoculant, *Pleurotus ostreatus*, animal feed

## Abstract

Distilled grain waste (DGW) is rich in nutrients and can be a potential resource as animal feed. However, DGW contains as much as 14% lignin, dramatically reducing the feeding value. White-rot fungi such as *Pleurotus ostreatus* could preferentially degrade lignin with high efficiency. However, lignin derivatives generated during alcohol distillation inhibit *P. ostreatus* growth. Thus, finding a new strategy to adjust the DGW properties to facilitate *P. ostreatus* growth is critical for animal feed preparation and DGW recycling. In this study, three dominant indigenous bacteria, including *Sphingobacterium thermophilum* X1, *Pseudoxanthomonas byssovorax* X3, and *Bacillus velezensis* 15F were chosen to generate single and compound microbial inoculums for DGW composting to prepare substrates for *P. ostreatus* growth. Compared with non-inoculated control or single microbial inoculation, all composite inoculations, especially the three-microbial compound, led to faster organic metabolism, shorter composting process, and improved physicochemical properties of DGW. *P. ostreatus* growth assays showed the fastest mycelial colonization (20.43 μg·g^−1^ ergosterol) and extension (9 mm/d), the highest ligninolytic enzyme activities (Lac, 152.68 U·g^−1^; Lip, 15.56 U·g^−1^; MnP, 0.34 U·g^−1^; Xylanase, 10.98 U·g^−1^; FPase, 0.71 U·g^−1^), and the highest lignin degradation ratio (30.77%) in the DGW sample after 12 h of composting with the three-microbial compound inoculation when compared to other groups. This sample was relatively abundant in bacteria playing critical roles in amino acid, carbohydrate, energy metabolism, and xenobiotic biodegradation, as suggested by metagenomic analysis. The feed value analysis revealed that *P. ostreatus* mycelia full colonization in composted DGW led to high fiber content retention and decreased lignin content (final ratio of 5% lignin) but elevated protein concentrations (about 130 g·kg^−1^ DM). An additional daily weight gain of 0.4 kg/d was shown in cattle feeding experiments by replacing 60% of regular feed with it. These findings demonstrate that compound inoculant consisting of three indigenous microorganisms is efficient to compost DGW and facilitate *P. ostreatus* growth. *P. ostreatus* decreased the lignin content of composted DGW during its mycelial growth, improving the quality of DGW for feeding cattle.

## 1 Introduction

Ruminant feed is the basis of animal husbandry. With the improvement in people’s living standards, the preference for meat products increases the demand for ruminant feed (Mizrahi et al., 2021). Conventional feed sources, such as corn, alfalfa grass, oat grass, and soybean meal, are insufficient in supply and quite expensive, limiting the development of livestock breeding, especially in underdeveloped areas. Thus, unconventional feed resources that do not compete with human nutrition, such as agro-industrial by-products of plant origin (e.g., distilled grains, oilseed waste, fruit and vegetable residues, sugar by-products, etc.) and agro-forestry waste (e.g., rice straw, coffee husks, cassava peels, cane trash, etc.) have attracted much attention from the feed industry and academia (Iram et al., 2020; Gupta et al., 2022).

Distilled grain waste (DGW), the primary by-product of the Chinese liquor industry, is composed of fermented grains, such as sorghum, wheat, corn, and rice husk (Tan et al., 2014). According to the National Bureau of Statistics, about 20 million tons of DGW are produced annually in China.^1^ Due to the high nutrient content, high acidity, and high moisture content, DGW is very easy to decay (Wang et al., 2017). Currently, the common DGW disposal methods include incineration, landfills, stacking, or fertilizer, but these methods cannot fully utilize the DGW, even causing serious environmental risks, such as unpleasant odor generation, soil and groundwater pollution (Wang et al., 2018).

DGW is enriched in sugars, proteins, fats, vitamins, and minerals, along with cellulose and hemicellulose, which are good energy sources for ruminants (Zhang et al., 2013). However, DGW contains approximately 14% lignin, a recalcitrant aromatic polymer encapsulating cellulose and hemicellulose, causing a significant reduction in cellulose and hemicellulose utilization efficiency by rumen microbes (Vanholme et al., 2019), thus reducing the feeding value of DGW. Various methods are available to degrade lignin, including physical, chemical, and biological methods. Physical methods, including soaking and grinding, have limited ability to degrade lignin (Li et al., 2022; Reshmy et al., 2022). Chemical methods, including alkalinization and ammonification, can cause serious environmental pollution and safety hazards in spite of the strong lignin degradation ability (Kamimura et al., 2019). Currently, biological methods using microorganisms have attracted much attention for the advantages of environmental friendliness, low energy consumption, and high efficiency. In particular, white-rot fungi, a category of microorganisms in nature that are effective at lignin degradation, have drawn much more attention (Wang J. et al., 2022). It has been pronounced by the degradation of various agro-industrial wastes, e.g., olive pruning residues, tea wastes, and spent coffee grounds (Abou Fayssal et al., 2021; Werghemmi et al., 2022). This not only reduces lignin dispersion in the environment but also helps in the bioremediation of various pollutants of anthropogenic sources (Širić et al., 2022).

*Pleurotus ostreatus*, one of the widely cultivated edible white-rot fungi worldwide, harbors a powerful enzymatic machinery to degrade lignin for carbon source and energy, retaining cellulose for rumen microbe utilization (Fernández-Fueyo et al., 2016; Zhao et al., 2020; Okuda et al., 2021). These ligninolytic enzymes are composed of lignin peroxidase (LiP), manganese peroxidase (MnP), laccase (Lac), exoglucanase (FPase), and xylanase (Fernández-Fueyo et al., 2016; Dissasa, 2022). In addition, the mycelia of *P. ostreatus* are a source of additional protein that can be used as animal feed without isolation (Krupodorova et al., 2024). *P. ostreatus* can be used to convert lignin in DGW to prepare high-quality animal feed. However, high concentrations of toxic phenolics in DGW (about 3.5 mg/g phenolic compounds) limit the rapid colonization of fungal hypha (Wu et al., 2021).

As an environmentally friendly biotechnology, composting is widely used as a pretreatment to accelerate fungal mycelial colonization (Vieira and de Andrade, 2016). Microorganisms trigger composting. An insufficient number of native microorganisms and the elimination of some functional microorganisms during succession may lead to low composting efficiency (He et al., 2022). Accordingly, to accelerate the composting process, researchers add functional microbial inoculants to the composting materials. For example, Wan et al. (2020) inoculated a mixture of strains isolated from chicken manure compost into a new compost pile to improve the efficiency of composting. Similar findings were reported by Xu et al. (2022). They observed that inoculating thermotolerant ammonia-oxidizing bacteria extended the sanitation stage and enhanced composting efficiency in cattle manure composting.

Previously, a consortium-based microbial agent consisting of five dominant indigenous bacteria, including *Sphingobacterium* sp. X1, *Ureibacillus* sp. X2, *Pseudoxanthomonas* sp. X3, *Geobacillus* sp. X4, and *Aeribacillus* sp. X5 was developed. The compound microbial inoculum exhibited potential application in DGW composting, providing substrates for *P. ostreatus* cultivation (Wu et al., 2021). To simplify and optimize the composition of the compound inoculant, *Sphingobacterium* sp. X1 (namely *Sphingobacterium thermophilum* X1 in this research) and *Pseudoxanthomonas* sp. X3 (*Pseudoxanthomonas byssovorax* X3), as well as the newly screened *Bacillus velezensis* 15F and *Caldibacillus hisashii* 22S, were selected in this study to evaluate the effects of single and mixed microbial inoculation on *P. ostreatus* colonization in composted DGW. The ergosterol content representative for fungal growth, the DGW physicochemical properties, and the microbial community dynamics and functional metabolism were analyzed. The lignocellulosic enzyme activity secreted by *P. ostreatus* and the following effect on the lignocellulose degradation were finally used to evaluate the composting efficiency treated with various inoculations for 12 h. The feed value was also assessed for the DGW inoculated with the three-microbial compound agent and incubated with *P. ostreatus* for 15 d and 30 d.

## 2 Materials and methods

### 2.1 Screening, identification, and culture of bacteria

The raw DGW from the thermophilic phase (about 55–60°C) of DGW composting was diluted, spread on LB agar plates (LB, tryptone 10 g·L^−1^, yeast extract 5 g·L^−1^, NaCl 10 g·L^−1^, Agar, 10 g·L^−1^), and then incubated at 55°C for strain isolation as previously described (Wu et al., 2021). The 16S rRNA gene of each strain was amplified using the primers Bact-27F (5 -AGAGTTTGATCMTGGCTCAG-3) and Bact-1492R (5 -GGTTACCTTGTTACGACTT-3) and sequenced. The alignment of these sequences was analyzed with the National Center for Biotechnology Information Database (NCBI; https://www.ncbi.nlm.nih.gov/Blast.cgi) (Yoon et al., 2017). The phylogenetic tree of the four chosen bacteria was constructed using the MEGA 7 program based on the maximum likelihood method with 1,000 bootstrap replicates (Kumar et al., 2016).

The four bacteria inoculum were prepared under optimized conditions as follows. For *S. thermophilum* X1 (ON965531) and *P. byssovorax* X3 (ON966119), the culture medium was composed of 10 g·L^−1^ molasses and 15 g·L^−1^ peptone, with an inoculum size of 1% and a culture temperature of 30°C. For *B. velezensis* 15F (ON970380) and *C. hisashii* 22S (OQ554990), the culture medium was composed of 5 g·L^−1^ molasses and 10 g·L^−1^ peptone, with an inoculum size of 1% and a culture temperature of 37°C.

### 2.2 Composting materials and processing

The materials for composting were DGW, corncob, and lime. Raw DGW was kindly provided by Anhui Golden Seed Winery Co., Ltd. (Anhui, China). It was cooled to room temperature and stored at 4°C for less than 2 weeks before use. Corncob and lime were purchased from Dezhou Fubang Agricultural Development Co., Ltd. (Shandong, China). The physicochemical properties of composting materials were listed in Supplementary Table S1. Corncob was crushed to approximately 5 mm in length and mixed with DGW in the ratio of 3:7 to adjust the C/N ratio to 25–35. The pH was adjusted to 6–7 with an addition of 4% lime (based on raw DGW weight), and the moisture content was adjusted to 60–70% with tap water (Bari et al., 2020; Wu et al., 2021).

Composting was carried out in a ventilated room, following the method described by Wu et al. (2021). Each pile was made of 200 kg of a mixture composed of DGW and corncob. For single microbial inoculation composting, the bacteria were cultured to the logarithmic growth phase, individually harvested by centrifugation, resuspended in sterile water, and added into DGW with an addition of 2% (v/m) at a cell density of approximately 1 × 10^8^ colony-forming units/mL. In compound microbial inoculation groups, the bacteria were mixed in equal proportions and diluted to the same volume as above. The Control Group CK was added with sterile water at 2% (v/m) final volume. Samples were collected at approximately 1 kg every 12 h or 24 h. Three samples were randomly taken from each pile at each time point. Piles were turned before each sampling to ensure aerobic conditions and the uniformity of the materials. Each sample was divided into three parts. One was stored at 4°C for analysis of pH, moisture content, electrical conductivity (EC), and germination index (GI), another was air-dried for *P. ostreatus*’s flask-cultivation and analysis of organic matter (OM) and total Kjeldahl nitrogen (TKN), and the last one was stored at −20°C for bacterial community and metabolism analysis.

### 2.3 *P. ostreatus* colonization and culture, and ergosterol content determination

*P. ostreatus* was maintained on PDA (potato dextrose agar, filtrate of boiled potato 200 g·L^−1^, glucose 20 g·L^−1^, agar 15 g·L^−1^) slants at 4°C. Four mycelial blocks (5 mm diameter) of actively grown *P. ostreatus* were inoculated into liquid PDA medium and incubated at 25°C for 5 d with shaking at 150 rpm. Then, a homogenizer was used to mix the mycelium as the seed. The DGW materials sampled from different composting groups were adjusted to pH 7.0 and 65% humidity, followed by sterilization, and used as the substrates. The seed of *P. ostreatus* was inoculated at 5% (v/m) into DGW substrates and incubated at 25°C and 60% humidity in flasks. Ergosterol was extracted by saponification reaction after 7 d of *P. ostreatus* colonization using the method described by Wu et al. (2021). The ergosterol content was analyzed via high-performance liquid chromatography method with an XDB C18 column (250 mm × 4.6 mm, 5 μm; Agilent, Palo Alto, United Stated) and a UV detector (1,260 DAD) at 30°C. Methanol was used as the eluting buffer, and 1.0 mL/min was set as the flow rate.

The 12 h composted DGW of different groups was used as the substrates to prepare wrapped bags (about 1.5 kg). The *P. ostreatus* seed was inoculated into the bags and cultured in a humidity-, temperature-, and light-controlled production house. Bags from different groups were withdrawn every 5 d for the determination of lignocellulose contents and enzyme activity. For DGW inoculated with the three-microbial compound agent, bags were withdrawn when the substrates had half mycelia growth, full mycelia growth, and after a round of mushroom harvesting for feed value evaluation. The DGW without *P. ostreatus* seeding was used as the control.

### 2.4 Physicochemical parameters analysis

Temperature was measured at the center of piles every 12 h with an electronic thermometer. The moisture content was measured after drying fresh samples at 105°C. The EC and pH were determined by mixing fresh samples with deionized water at a ratio of 1:10 (m/v). After shaking for 0.5 h, the mixture was filtered to obtain the supernatant. The weight loss after ignition at 550°C for 4 h in a muffle furnace was used to determine the OM content (Lu et al., 2018). In accordance with the Chinese standard GB/T 6432–2018, TKN was measured. Following the method described by Qian et al., GI was detected (Qian et al., 2022).

### 2.5 Bacterial community and function analysis

Total genomic DNA was extracted from 12 h composted DGW samples of different groups using the DNeasy PowerSoil Kit (Qiagen, Germany), according to the manufacturer’s instructions. Then, DNA was determined after Nanodrop checking. Universal primers (338F: 5 -ACTCCTACGGGAGGCAGCAG-3, 806R: 5 -GGACTACHVGG GTWTCTAAT-3) were performed to amplify V3-V4 region of the bacterial 16S rRNA gene. The amplicon libraries were constructed and sequenced on the MiSeq PE250 sequencer (Illumina, United States) at Shanghai Personal Biotechnology Co., Ltd. (Shanghai, China). Chao1, Shannon, and microbial community graphs were performed using the genescloud tools.^2^ Bacterial composition and distributive abundance in the samples were conducted at the genus level using QIIME2 2019.4 and R packages (vision 3.2.0) based on the sequence data and visualized using MEGAN and GraPhlAn as previously described (Wu et al., 2021). Microbial functions were predicted using the software PICRUSt2 and drawn in a heatmap using the “pheatmap” package of the R software (version 3.6.3) (Kolde and Kolde, 2015). The correlation network diagram was drawn with Cytascape software (version 3.10.1) (Gong et al., 2023).

### 2.6 Determination of lignocellulose contents and enzyme activities during *P. ostreatus* cultivation

DGW substrates were sampled from the wrapped bags of *P. ostreatus* culture every 5 days during the first month for lignocellulose contents and enzyme activity determination. Three bags were withdrawn for each group at a time. The concentrations of cellulose, hemicellulose, and lignin were analyzed using the method described by Soest et al. (1991). Enzymes were extracted as described by Zeng et al. (2010). Lac activity was determined by 2, 2-azino-bis (3-ethylbenzothiazoline-6-sulfonic acid) (Couto et al., 2006). MnP activity was measured by the 2, 6-dimethylphenol (2, 6-DMP) method (Wariishi et al., 1992). The veratryl alcohol was used to determine Lip activity (Arora and Gill, 2001). Xylanase activity was determined by xylan solution and 3, 5-dinitrosalicylic acid (DNS) (Irfan et al., 2016). Whatman No. 1 filter paper strip (1 × 6 cm) and xylan solution were used as substrates for the determination of filter paperase (FPase) and xylanase activities, respectively (Zhao et al., 2021).

### 2.7 Determination of chemical composition in composted DGW samples

About 100 g samples were analyzed for dry matter (DM) content by drying them in a forced-air oven at 65°C for 48 h and then grinding to pass a 1.0 mm screen for chemical analysis. Crude protein (CP) was measured according to the methods of the Association of Official Analytical Chemists (AOAC, 1990) using a Kjeldahl nitrogen analyzer (SKD-100, Shanghai Peiou Analytical Instrument Co., LTD, China). Protein fractions, including true protein (TP), nonprotein-N (NpN), and free amino acid (FAA) were determined according to the method of Licitra et al. (1996). The neutral detergent fiber (NDF) and acid detergent fiber (ADF) contents were detected as described by Soest et al. (1991) without the use of heat-stable amylase and sodium sulphite by an XD-CXW-10 Fiber Analyzer (Shanghai Zida Instrument Co., LTD, China).

### 2.8 Cattle feeding

A cattle feeding experiment was conducted at the Livestock and Poultry Breeding Service Center of Fuyang City (Anhui, China). The protocol involving animals was approved and carried out strictly following the related regulations (Hefei, China).

The DGW substrates from Group C4 with full mycelia growth were collected together. For the safety and adaptation of cattle, DGW feed was mixed with regular feed. The total daily intake of each cattle was 18 kg, and feeding times were 4 am and 4 pm, respectively. Thirty cattle were divided into three groups. The feeding experiment lasted 4 weeks, and the weight of cattle was recorded. The feed of Group A was 100% regular feed, Group B was composed of 70% regular feed and 30% DGW feed, and Group C was composed of 40% regular feed and 60% DGW feed.

### 2.9 Statistical analysis

GraphPad Prism 9 was used for physicochemical, lignocellulose, enzymes, and chemical composition analyses. ChiPlot^3^ was used for ergosterol analysis. The statistical significance was evaluated through one-way ANOVA, followed by Student’s *t*-test with GraphPad Prism 9.0. The significance standard was *p* value <0.05. All experiments were conducted with three biological replications except for the cattle feeding experiment with 10 replications.

## 3 Results and discussion

### 3.1 Evaluation of the individual and combined inoculation effects of indigenous bacteria in DGW compost to accelerate *P. ostreatus* colonization

*Sphingobacterium*, *Pseudoxanthomonas*, *Bacillus*, and *Caldibacillus* are effective decomposers involved in the degradation of organic matter during various substrate composting (Neelkant et al., 2019; Wu et al., 2020; Chang et al., 2023; Li et al., 2023; Sun et al., 2023), occupying the four highest abundance for species richness during DGW composting (Wu et al., 2021). Each strain of these genera, including the strains *S. thermophilum* X1 and *P. byssovorax* X3 used previously (Wu et al., 2021), and the newly isolated ones *B. velezensis* 15F and *C. hisashii* 22S were selected for further single and compound microbial inoculated composting experiments (Supplementary Figure S1).

Firstly, DGW composting inoculated by individual strain was carried out. The samples were collected every 24 h and used as substrates for *P. ostreatus* colonization. Ergosterol content was chosen as a marker of fungal growth and colonization rate of *P. ostreatus* (Mansoldo et al., 2020; Wu et al., 2021; Wang Q. et al., 2022). *P. ostreatus* could not grow when directly using the mixed raw DGW as substrates (Supplementary Figure S2). However, the ergosterol contents gradually increased in DGW samples composted for 0–48 h, suggesting decreased toxic substances and increased soluble nutrients (Yadav et al., 2020; Wu et al., 2021). DGW substrates treated by *S. thermophilum* X1 (named T1), *P. byssovorax* X3 (T2), and *B. velezensis* 15F (T3) were more suitable for *P. ostreatus* growth. Furthermore, T1 and T3 inoculation also accelerated the mycelial colonization since their ergosterol contents (20.51 and 18. 58 μg·g^−1^ in DGW composted for 48 h) peaked 24 h earlier than CK group (17. 48 μg·g^−1^ in DGW composted for 72 h). Conversely, *C. hisashii* 22S (T4) inoculation did not affect *P. ostreatus* growth. The ergosterol contents between Groups T4 and CK were almost similar during the whole composting process (Supplementary Figure S2).

**Figure 1:**
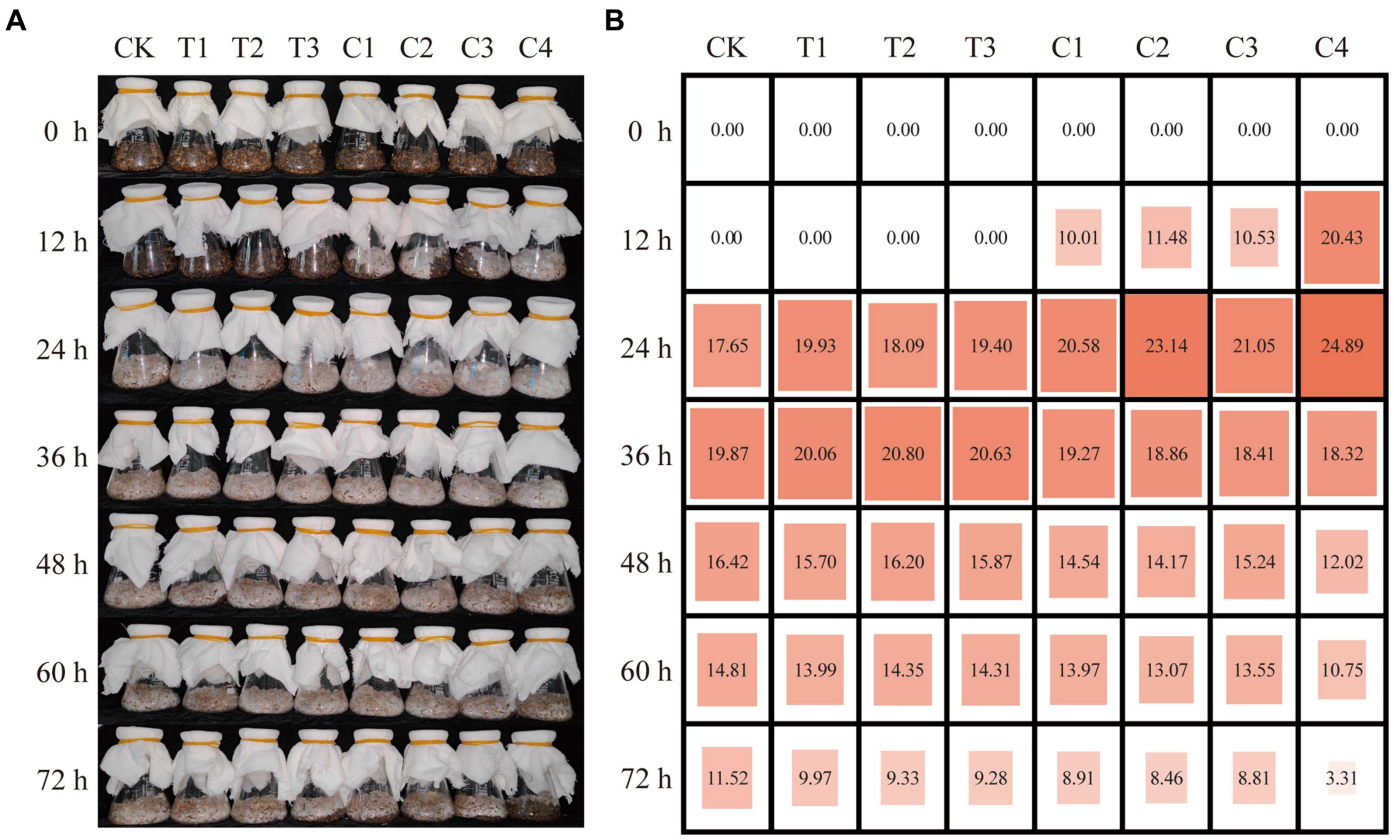
The effects of individual and combined inoculants on DGW compost to accelerate *P. ostreatus* colonization. Growth **(A)** and the ergosterol content (μg·g^−1^) **(B)** of *P. ostreatus* colonized in DGW inoculated with individual or combined inoculums every 12 h of composting.

Thus, T1, T2, and T3 were further selected and combined to generate four composite microbial inoculants, including C1 (T1 and T2), C2 (T1 and T3), C3 (T2 and T3), and C4 (T1, T2, and T3). Another composting experiment was performed, and composting settings were named Groups T1-T3 and C1-C4 according to inoculated microbial agents, respectively. Samples were taken every 12 h to compare the effects of single and compound microbial inoculated DGW compost on *P. ostreatus* growth. In comparison with no detected ergosterol content in DGW treated with single microbial inoculants and composted for 12 h, *P. ostreatus* could grow well in DGW inoculated with composite microbial inoculants (Figure 1). Among them, the highest ergosterol content was detected in Group C4 (20.43 μg·g^−1^), about twice that of the Groups C1 to C3 (10.01 μg·g^−1^, 11.48 μg·g^−1^, and 10.53 μg·g^−1^, respectively). The mycelial colonization was accelerated in composite inoculum treated groups by 12 h as compared with the single microbial treated groups, with the peaked ergosterol contents in DGW composted for 24 h at 20.58, 23.14, 21.05, and 24.89 μg·g^−1^, respectively, in Groups C1 to C4 (Figure 1B). More importantly, the ergosterol content in Group C4 which used DGW composted for 12 h as the substrate was comparable to the peaked ones in other groups.

### 3.2 The three-microbial compound inoculum harbors the best effect on the improvement of physicochemical properties of DGW compost

**Figure 2:**
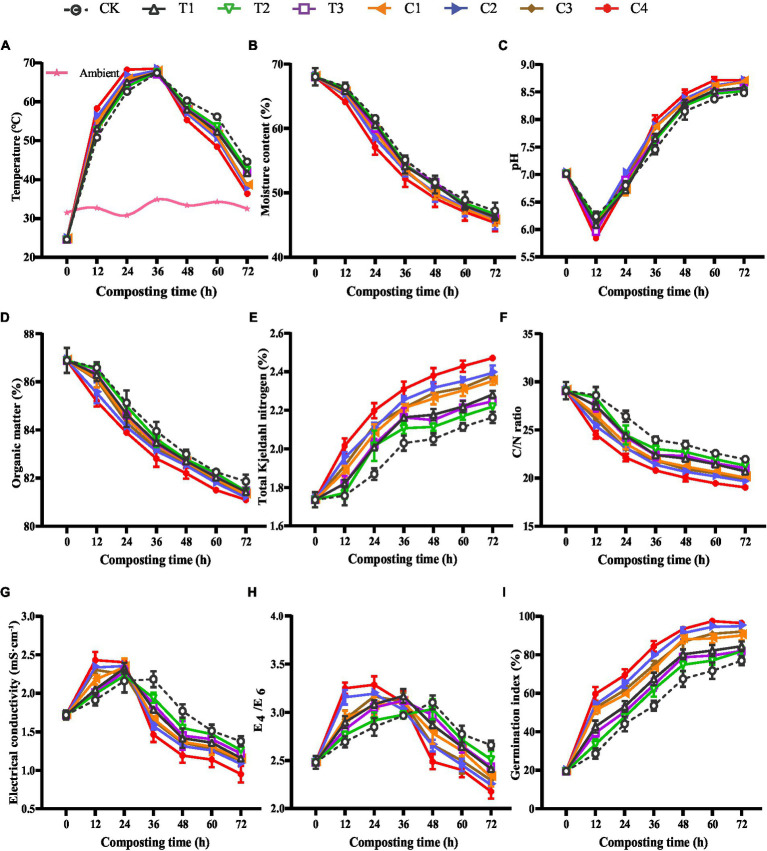
The physicochemical properties of DGW composting with individual or combined inoculations. **(A)** Temperature. **(B)** Moisture content. **(C)** pH. **(D)** Organic matter. **(E)** Total Kjeldahl nitrogen. **(F)** C/N ratio. **(G)** Electrical conductivity. **(H)** E_4_/E_6_. **(I)** Germination index.

The physicochemical properties of DGW compost were analyzed for each efficient group every 12 h to explore the mechanism promoting *P. ostreatus* colonization. The DGW composting process could be divided into mesophilic, thermophilic (> 50°C), and cooling phases (Wu et al., 2021). As shown in Figure 2A, all microbial inoculations accelerated the composting process, suggesting more active microbial metabolism and faster degradation of organic matter (Yang and Zhang, 2022). The groups entered the thermophilic phase following the order of C4 > C2 > C3 > C1 > T1 > T3 > T2 > CK (at 12 h of composting). Compared to Group CK, inoculated groups had higher temperatures in mesophilic and thermophilic phases at the same composting time. The temperature of DGW treated with C4 reached the peak of 68.3°C at 24 h of composting, whereas the other groups had the highest temperatures with a range from 67.0°C to 68.0°C at 36 h of composting. The acceleration of composting temperature is essential for the rapid killing of potential pathogens, such as *Enterobacter* and *Acinetobacter*, in the raw DGW, as well as for the compost hastening maturity (Joseph et al., 2018). Following this fact, the temperatures in microbial inoculated groups decreased faster than Group CK after 36 h of composting, with Group C4 entering the cooling phase most quickly (Figure 2A), perhaps due to the fastest consumption of soluble and readily assimilable compounds (Chan et al., 2016; Zhao et al., 2016; Sánchez et al., 2017).

The active metabolism during DGW composting was also reflected by the continuously decreased moisture content in all groups (Figure 2B). Consistent with the order of temperature rising rate that was responsible for moisture evaporation, the degree of moisture content reduction was in the order of C4 > C2 > C3 > C1 > T1 > T3 > T2 > CK. Similar results were also found in other compostings, such as organic–inorganic aerobic composting and chicken manure composting with maize straw (Yu et al., 2019; Wan et al., 2020). Group C4 showed the fastest drop in moisture content (Figure 2B), suggesting the maximum moisture dissipation caused by most active microbial metabolism. At the initial stage, the high moisture content (68.03%) might let microorganisms utilize the organic matter to produce certain organic acids, such as lactic acid and butyric acid, in the anaerobic fermentation piles (Zhang et al., 2019), resulting in a temporary drop in pH to 5.6–6.2 after 12 h of composting in each group (Figure 2C). After this period, all groups maintained a steady increasing pH in the thermophilic phase and stabilized at a pH of about 8.5 in the cooling phase. The pH values of microbial inoculated groups, especially Group C4, not only decreased faster during the first 12 h of composting but also rose faster later than that of the CK group (Figure 2C).

The increased pH was attributed to the degradation of organic acids and the utilization of nitrogenous organic matter and ammonification by microorganisms (Yang et al., 2020). Compared with Group CK, the OM decreased faster in microbial-inoculated groups during the DGW composting process (Figure 2D), suggesting a stronger depletion of OM (Zhang et al., 2019; Duan et al., 2020). The degradation of OM followed the order of C4 > C2 > C3 > C1 > T1 > T3 > T2 > CK. Correspondingly, the change of the TKN concentration was opposite among the eight groups because of the reduction of the compost mass caused by OM degradation (Figure 2E). The metabolism of carbon is often faster than nitrogen in composting studies using various substrates including DGW (Wu et al., 2020, 2021; Wang L. et al., 2022). Thus, the C/N ratio in Group C4 was the lowest among the eight groups, followed by two microbial inoculated groups (Figure 2F).

The rapid decomposition of various OM into small soluble molecular components such as organic acids and NH_4_^+^ by microorganisms and the reduced compost mass is assumed to lead to increased EC (Chen et al., 2020; Sun et al., 2023). In the mesophilic phase and early stage of the thermophilic phase, the EC values increased in all groups, with microbial inoculated groups higher than Group CK (Figure 2G). However, in the late stage of the thermophilic phase and the cooling phase, the volatilization of organic acids or NH_3_ and humification conversion resulted in decreased EC values (Bernal et al., 2009; Xu et al., 2021; Sun et al., 2023). This was also confirmed by the changes in E_4_/E_6_ value, which represented the condensation degree of the aromatic substances and indicated inverse proportion to the humification levels (Wan et al., 2020). The EC and E_4_/E_6_ in microbial inoculated groups not only decreased earlier but also harbored lower values than Group CK (Figures 2G,H). Therefore, the inoculation of microorganisms reinforced both the microbial metabolism and humic substance formation in DGW compost. Among the composite inoculants, C4 worked the most efficiently.

The GI value can be used as an indicator of the toxicity of a sample (Kong et al., 2022). With the degradation of toxic substances such as organic acids, alcohols, and aldehydes, and increased humification, the GI value showed an increasing trend (Figure 2I). Group C4 had a GI value of 69.2% after 24 h of composting, in comparison with 44.1% in Group CK. Whereas after 48 h of composting, the GI value rose to about 95% in Group C4, indicating the almost total degradation of toxic substances and the maturation of compost (Xu et al., 2021; Sun et al., 2023). Meanwhile, the GI values in Group CK and three single-microbial inoculated groups were lower than 80%. These results in total suggested that the physicochemical properties of DGW compost were improved by microbial inoculation, and the microbial inoculant C4 consisting of T1, T2, and T3 was an effective compound agent for DGW composting.

### 3.3 The three-microbial compound inoculum changes the bacterial community dynamics and functional metabolism in DGW compost

**Figure 3:**
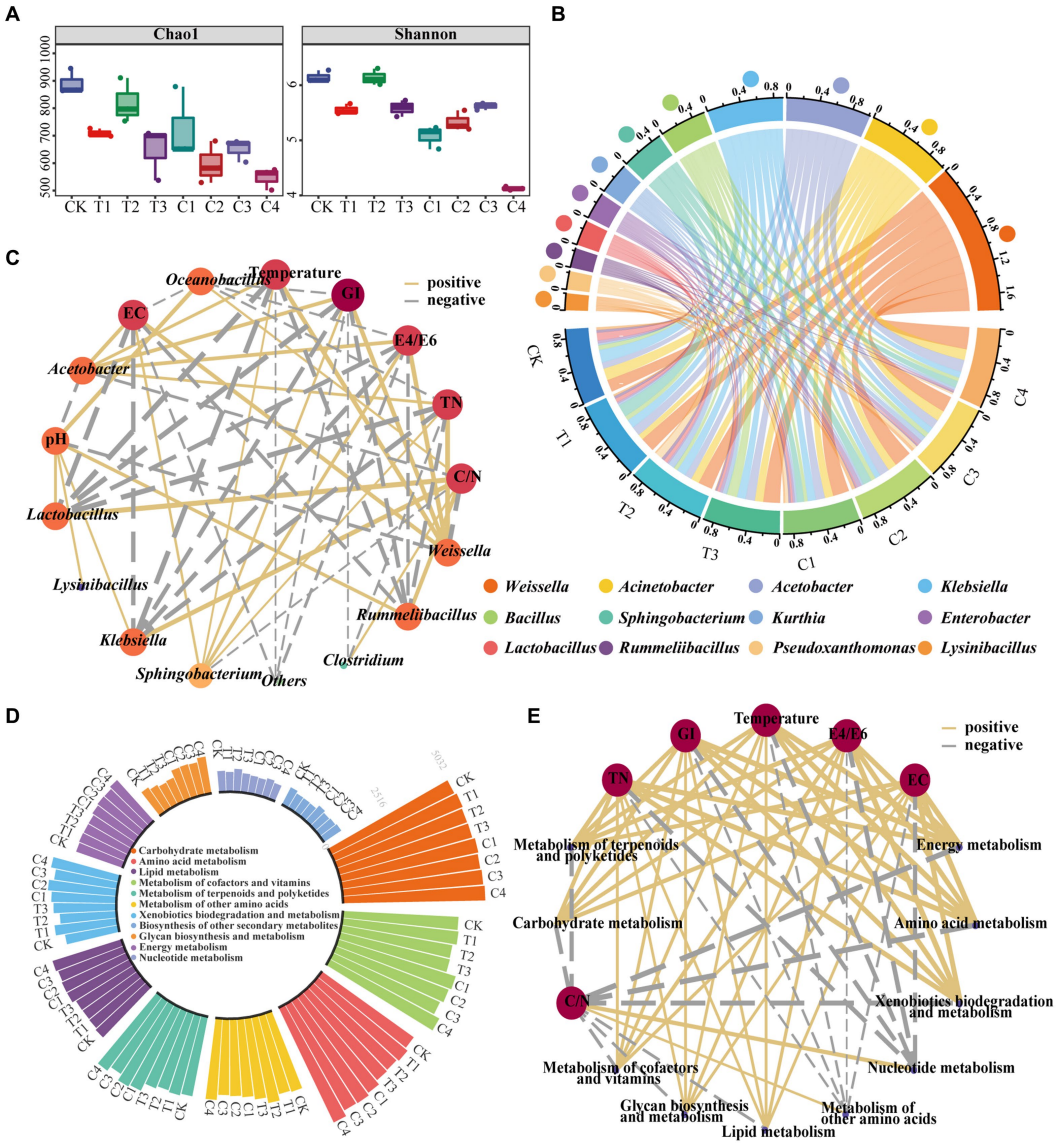
Changes in bacterial community composition and potential functions in composted DGW samples. DGW samples were treated with individual and combined inoculations and composted for 12 h. **(A)** Changes in the Chao1 index and Shannon index. **(B)** Variations of the microbial community at the genus level. **(C)** The correlation network diagram between bacterial communities and physicochemical properties of DGW. **(D)** Variations of microbial metabolic functions. **(E)** The correlation network diagram between bacterial functions and physicochemical properties of DGW.

Bacterial community succession is an intrinsic factor in changing the physicochemical properties of DGW (Chen X. et al., 2022; Qv et al., 2023). The DGW samples composted for 12 h, in which the growth rates of *P. ostreatus* dramatically differed among groups inoculated with different types of bacteria (Figure 1), were chosen for 16S rDNA sequencing. Compared to CK, the Chao1 index and Shannon index suggested that the richness and diversity of the microbial community decreased in almost all microbial inoculated groups (Figure 3A). The lowest Chao1 and Shannon indexes were both observed in Group C4 (Figure 3A). Therefore, microbial inoculation addition led to the enrichment of superior microorganisms in DGW compost. These alterations were consistent with swine manure and rice straw co-composting with *Streptomyces griseorubens* inoculation (Chi et al., 2020) but inconsistent with sewage sludge composting inoculated with a compound bacteria agent (Chen X. et al., 2022). According to Figure 2A, microbial inoculations have driven the composting process to the thermophilic phase at 12 h of composting. The heat might be responsible for decreased microbial richness and diversity. Furthermore, the non-metric multidimensional scaling (NMDS) analysis suggested that the microbial communities were distant from each other among groups (Supplementary Figure S3), in line with the altered DGW properties.

After composting for 12 h, *Weissella, Acinetobacter, Acetobacter, Klebsiella,* and *Bacillus* were the top 5 dominant genera. *Weissella*, a genus of lactic acid bacteria, plays a key role in promoting nitrogen cycling and accelerating composting (He et al., 2022; Jin et al., 2022; Lu et al., 2023). Microbial inoculants for each group stimulated the growth of *Weissella*. Among, the highly significant difference in the relative abundance of *Weissella* between Group CK (17.02%) and C4 (30.73%) may account for the fastest fermentation in Group C4 (Figure 3B; Supplementary Figure S4; *p* < 0.001). Similarly, the highest relative abundance of *Acetobacter* was found in Group C4, probably related to the fact that it degraded the most alcohol in DGW samples (El-Askri et al., 2022; Matsumoto et al., 2023). On the contrary, the relative abundance of *Klebsiella*, a genus of pathogenic bacteria, was lower in microbial inoculated groups compared to CK, especially in Groups C2-C4 (Figure 3B; Supplementary Figure S4), probably because of their higher temperature at 12 h of composting than CK to kill more pathogens. Another genus, *Acinetobacter*, is essential for organic matter degradation during composting (Wu et al., 2022), but no significant difference was observed among groups. The abundance of *Bacillus*, which is widely found in the composting process and plays a vital role in the degradation of organic matter (Zhang et al., 2021), was balanced in the remaining groups, except for Group T3 which was inoculated with a relatively high number of *B. velezensis* 15F. Furthermore, the relative abundance of *Sphingobacterium* in C1, C2, and C4 groups treated with *S. thermophilum* X1 was higher than that of other groups (Supplementary Figure S4). A similar phenomenon was observed for the genus *Pseudoxanthomonas*. These results indicated that inoculated microorganisms survived during DGW composting and correlated with bacterial community dynamics, temperature changes, and organic matter degradation in composting since *Pseudoxanthomonas* has been demonstrated to degrade environmental hydrocarbons and *Sphingobacterium* is a biomarker for organic biodegradation strengthening toward humification (Mohapatra and Phale, 2021; Qi et al., 2022).

Next, the correlation network diagram between bacterial communities and physicochemical properties of DGW was further performed with Cytascape software. Thirty-three paired correlations were obtained [| r | > 0.6, *p* < 0.05], among which *Weissella* and *Rummeliibacillus* having a large number of connections with DGW physicochemical properties. As shown in Figure 3C, *Weissella* and *Sphingobacterium* abundance was positively correlated with temperature, TN, EC, E4/E6, and GI, but negatively correlated with C/N. Conversely, the abundance of *Klebsiella*, *Rummeliibacillus* and *Lactobacillus* was positively correlated with C/N, but negatively correlated with temperature, TN, EC, E4/E6, and GI (Figure 3C). The temperature was also positively associated with other genera including *Acinetobacter* that are thermophilic bacteria and harbor strong degradation abilities of carbohydrates and cellulose (Karthika et al., 2020; Gao et al., 2021; Cazaudehore et al., 2022).

High levels of microbial activity are always associated with increased microbial metabolism (Zhou et al., 2019; Zhong et al., 2020). After inoculation, the levels of amino acid, carbohydrate, energy, cofactors, and vitamins metabolisms in Groups T1 and C1-C4 were higher than in the CK group (Figure 3D). The xenobiotic biodegradation and metabolism, as well as terpenoid and polyketide metabolism, was accelerated in inoculated groups except for Group T2. Among, Group C4 showed the highest metabolism levels of the six metabolic functions, which may be related to the strongest depletion of complex organic matter in DGW, including carbohydrates, alcohols, phenols, and aldehydes, and the fastest increase in temperature (Figure 3D). In addition, the glycan biosynthesis and metabolism was much higher in Group C4 than other groups, in consistency with the higher ergosterol content of Group C4. Relatively, Group T2 had the highest metabolism of other amino acids and nucleotide metabolism. The correlation network diagram between metabolic functions and physicochemical properties of DGW showed 60 paired correlations [| r | > 0.6, *p* < 0.05]. The correlation between GI and temperature tended to be consistent. Both of them were positively associated not only with the metabolism of amino acids, carbohydrates, lipids, energy, cofactors and vitamins, and terpenoids and polyketides but also with glycan biosynthesis and metabolism and xenobiotics biodegradation and metabolism (Figure 3E). Therefore, the three microbial compound inoculum could drive the bacterial community dynamics and metabolism change, upregulate the composting temperature, and improve DGW physicochemical properties, ultimately facilitating *P. ostreatus* growth.

### 3.4 *P. ostreatus* decreases the lignin content and improves the feed value of composted DGW

**Figure 4:**
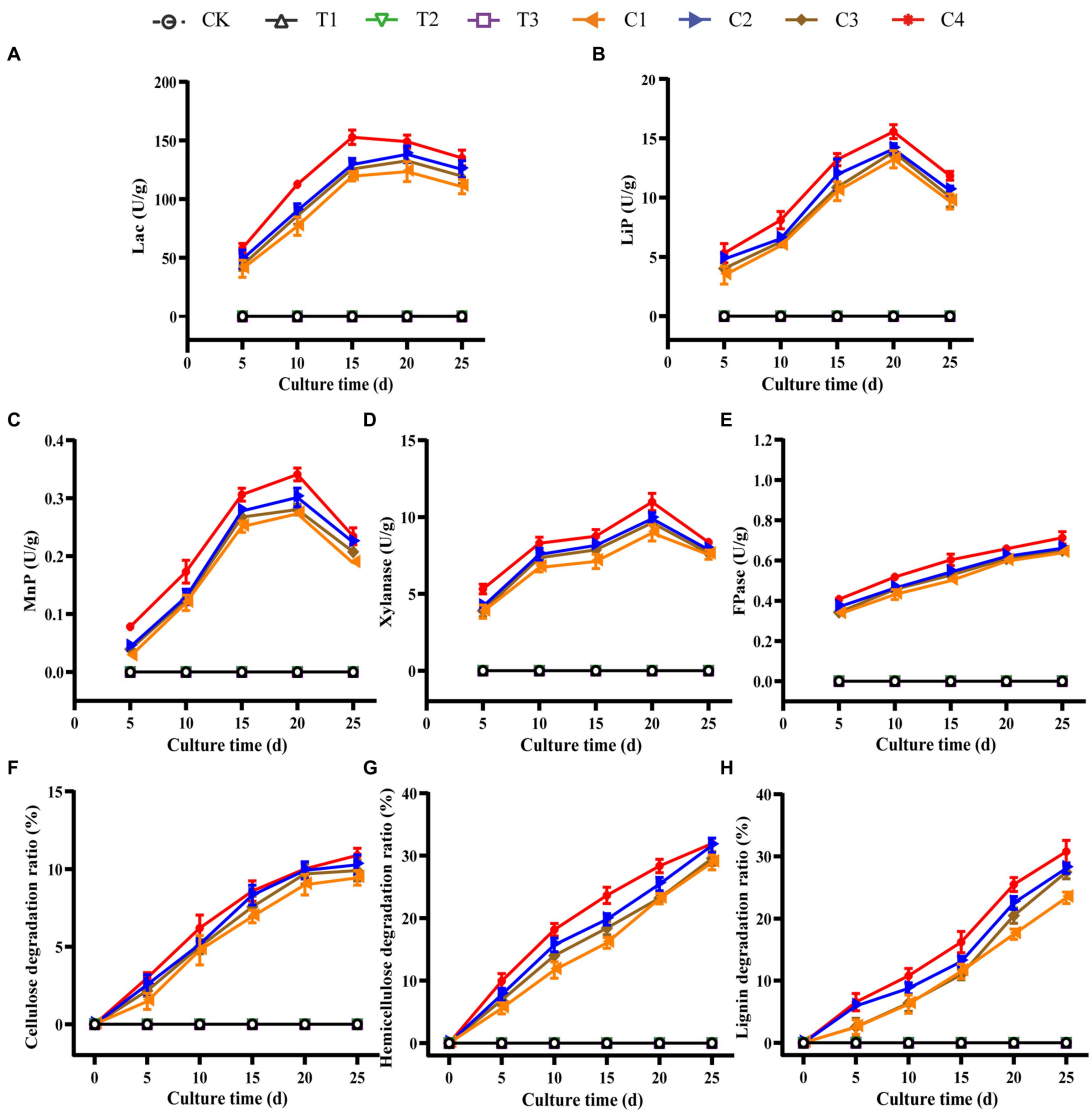
Dynamic changes of the lignocellulose degradation and relative enzymatic activities in substrate bags. DGW materials treated by microbial inoculums and composted for 12 h were used as substrates to wrap into bags. *P. ostreatus* mycelia were inoculated to culture mushrooms. The substrates were withdrawn every 5 d to detect activities of Lac **(A)**, LiP **(B)**, MnP **(C)**, xylanase **(D)**, FPase **(E)**, and the degradation ratios of cellulose **(F)**, hemicellulose **(G)**, and lignin **(H)**.

DGW materials treated with microbial inoculums and composted for 12 h were withdrawn and wrapped into substrate bags (about 1.5 kg) to cultivate *P. ostreatus*. The lignocellulose degradation and relative enzymatic activities in DGW were measured every 5 d to compare the mycelial growth status. There was no lignocellulosic enzyme activity and lignocellulose degradation in Groups CK and T1 to T3 (Figures 4A–H), in line with the absence of ergosterol in these groups (Figure 2B). However, the corresponding enzyme activities and degradation ratios of cellulose, hemicellulose, and lignin in all compound microbial inoculated groups were substantially enhanced. Group C4 had the highest LiP, MnP, Lac, FPase, and Xylanase activities (Figures 4A–E) in association with the highest degradation of lignocellulose (Figures 4F–H). Specifically, Lac activity in Group C4 peaked at 15 d with 152.68 U·g^−1^, LiP activity peaked at 20 d with 15.56 U·g^−1^, MnP activity was the highest at 20 d with 0.34 U·g^−1^, Xylanase activity was the highest at 20 d with 10.98 U·g^−1^, and FPase activity continued to increase to 0.71 U·g^−1^ at 25 d. The degradation ratios of cellulose and hemicellulose were similar between microbial inoculated groups; for example, Group C4 harbored 10.88% cellulose degradation (Figure 4F) and 31.93% hemicellulose degradation (Figure 4G). The highest level of degradation in lignin at 25 d was observed in Group C4, with a 30.77% degradation ratio and higher than other compound microbial inoculated groups (Figure 4H). In addition, the mycelial extension speed in the bag in Group C4 was substantially accelerated, reaching 9 mm/d, as compared to about 6 mm/d in Groups C1–C3.

The DGW substrates from Group C4 with half mycelia growth, full mycelia growth, and after a round of mushroom harvesting were evaluated for their feed value. In accordance with the conclusion that *P. ostreatus* had effective and widespread applications in lignin removal from various by-products for animal feed, such as cornstalk (Chen et al., 2017) and purple field corn stover (Khonkhaeng and Cherdthong, 2019), the final lignin ratio decreased from 14% in raw DGW to about 12%, 5%, and 5%, respectively. As shown in Table 1, the content of DM in the substrate decreased gradually following mycelial growth. In contrast, the contents of CP and TP increased, with an 11.5% and a 36.5% increment in the substrate with half mycelia growth and a 42.3% and a 53.6% increase in the substrate with full mycelia growth as compared with DGW without mycelial colonization, respectively. After a round of harvesting, the total protein concentrations in the substrate decreased to levels comparable to the DGW without mycelial colonization. Furthermore, all substrates tested were high in fiber content (736.3 and 441.9 g/kg DM for NDF and ADF in DGW without mycelia colonization, 730.0 and 449.3 g·kg^−1^ DM for NDF and ADF in the DGW with half mycelia growth, and 725.9 and 442.8 g·kg^−1^ DM for NDF and ADF in the DGW with full mycelia growth, respectively, in Trail 1). Hence, *P. ostreatus* mycelia colonized DGW substrate, particularly when fully grown, maintained a high fiber content while increasing protein levels and reducing lignin content. This value indicates its potential use in preparing ruminant feed (Zebeli et al., 2012; Erickson et al., 2020; Chen L. et al., 2022). In addition, compared with the DGW without *P. ostreatus* culture, the water soluble carbohydrate in the DGW with full mycelia growth was slightly higher whereas the free amino acid content was slightly lower.

**Table 1:** Chemical compositions of the Group 4 DGW substrates with different states of mycelial growth.

	Trial 1				Trial 2			
	Before	Half	Full	After	Before	Half	Full	After
DM (g·kg^−1^)	463.0	356.9	343.4	372.0	464.0	366.5	348.5	374.0
CP (g·kg^−1^ DM)	91.0	101.5	129.5	87.5	87.5	103.3	131.3	99.3
TP (g·kg^−1^ DM)	71.8	98.0	110.3	73.5	70.0	98.0	105.0	77.0
NpN (g·kg^−1^ DM)	3.1	0.6	3.1	2.2	2.8	0.8	4.2	3.6
NDF (g·kg^−1^ DM)	736.3	730.0	725.9	739.0	735.3	731.4	706.1	688.6
ADF (g·kg^−1^ DM)	441.9	449.3	442.8	463.2	447.4	470.0	491.8	438.0
WSC (g·kg^−1^ DM)	41.6	37.4	36.4	48.3	38.6	43.7	28.7	44.7
FAA (mg·100 g^−1^ DM)	33.4	70.7	41.7	25.3	33.0	69.0	44.5	26.4

Before, DGW without mycelial colonization; Half, DGW with half mycelia growth; Full, DGW with full mycelia growth; After, DGW after a round of mushroom harvesting. DM, dry matter; CP, crude protein; TP, true protein; NpN, nonprotein nitrogen; NDF, neutral detergent fiber; ADF, acid detergent fiber; WSC, water soluble carbohydrate; FAA, free amino acid.

### 3.5 The composted DGW with full mycelial growth is a favorable ruminant feed

**Figure 5:**
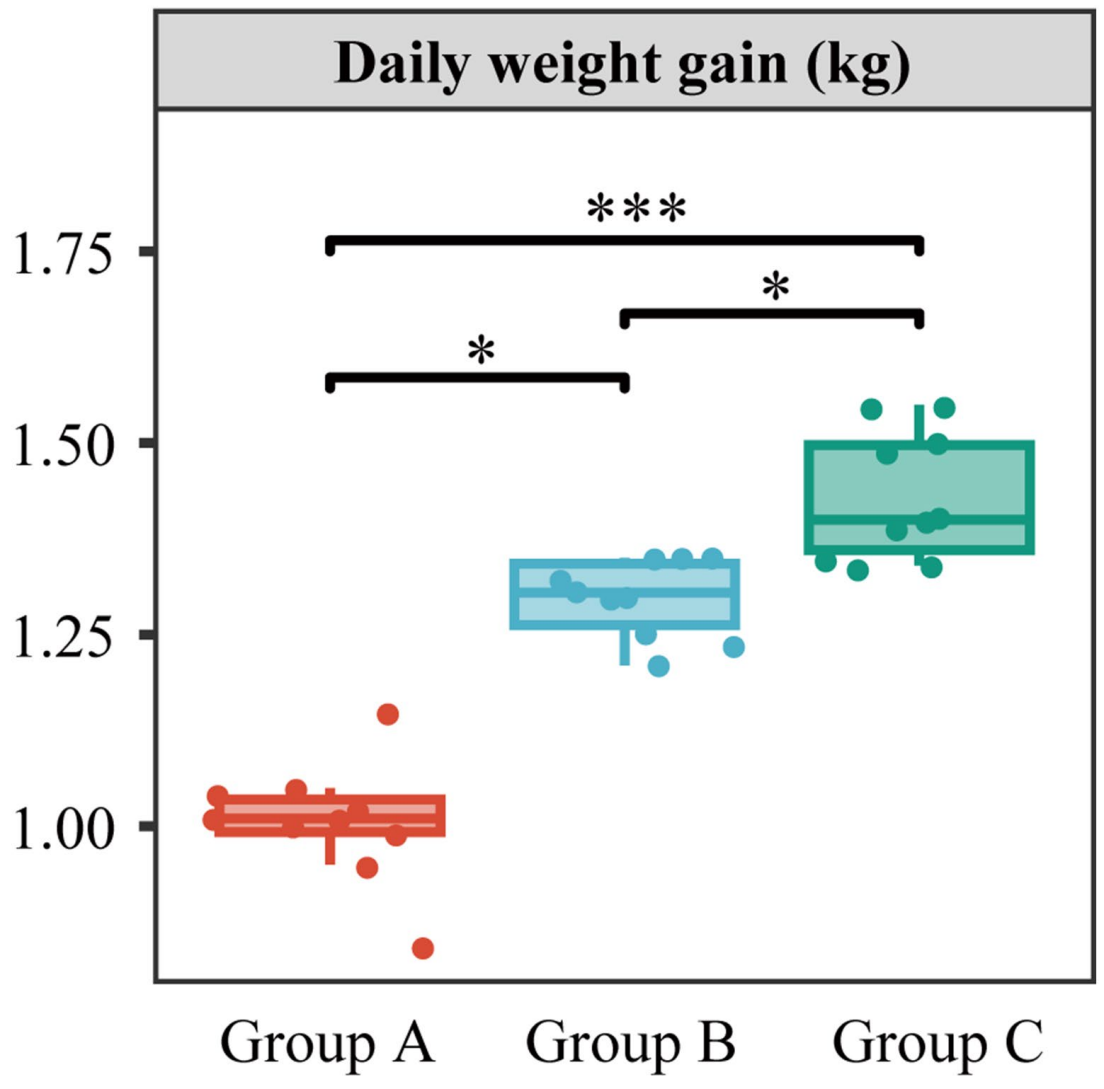
Daily weight gain of cattle using DGW feed. Cattle in groups A, B, and C were given feed including 0, 30, and 60% DGW, respectively. (significant statistical differences: * *p* < 0.05, *** *p* < 0.001). Data present means ± SD, *n* = 10.

DGW from Group C4 with full mycelial growth was further collected for animal feeding studies. Three batches of cattle feeding experiments were performed to compare the effect of different amounts of DGW addition on weight gain (Figure 5). Cattle in group C (60% DGW feed and 40% regular feed) had a daily weight gain of 1.4 kg, followed by 1.3 kg in group B (30% DGW feed and 70% regular feed) and 1.0 kg in group A (100% regular feed). This result indicated that DGW, after fermentation with the compound microbial inoculum C4 followed by *P. ostreatus* culture, is a favorable ruminant feed. This feed is composed of the physical and chemical properties-optimized DGW, fungal mycelia, and the extracellular enzymes and bioactive compounds secreted from mycelia (Antunes et al., 2020). These substances provide high nutritional value for ruminants.

## 4 Conclusion

Three strains were employed and inoculated in DGW in different combinations to explore a new recycling strategy of DGW. After pretreatment of DGW with the compound microbial inoculant consisting of three effective strains, the physicochemical properties of DGW were mostly improved through driving by the changes of microbial community structures and functions. The *P. ostreatus* colonization and mycelial growth were substantially faster, resulting in decreased lignin content and increased protein concentrations in the substrate. Cattle feeding using the composted DGW with full mycelial growth leading to more weight gain further reveals its potential application in ruminant feed. Therefore, a new approach comprised of microbial inoculated composting, *P. ostreatus* culture, and followed by animal feeding is suitable and highly valued for DGW cycling. Subsequent studies can be proposed based on this study to combine *P. ostreatus* culture and other strategies to elevate the protein content further, optimize the feed value, and prolong the storage period.

## Data availability statement

The raw sequencing data were uploaded to the National Center for Biotechnology Information (NCBI) Sequence Read Archive (SRA) database with the accession number PRJNA917117.

## Ethics statement

The animal study was approved by the ethical and humane committee of Anhui University. The study was conducted in accordance with the local legislation and institutional requirements.

## Author contributions

LY: Data curation, Investigation, Writing – original draft, Conceptualization. ZA: Data curation, Investigation, Writing – original draft, Methodology. DX: Investigation, Writing – original draft. DY: Investigation, Writing – original draft. GX: Methodology, Resources, Writing – review & editing. XG: Methodology, Resources, Writing – review & editing. YX: Writing – review & editing. JL: Writing – review & editing, Conceptualization, Data curation, Supervision, Writing – original draft. ZF: Conceptualization, Supervision, Writing – review & editing, Funding acquisition.

## Footnotes

^1^https://data.stats.gov.cn

^2^https://www.genescloud.cn/

^3^https://www.chiplot.online/
