# Supplementary material for: From waste to protein: a new strategy of converting composted distilled grain wastes into animal feed
Source: Front Microbiol. 2024 May 31;15:1405564. doi: 10.3389/fmicb.2024.1405564 (PMC11176434; doi:10.3389/fmicb.2024.1405564)
Supplement: Supplementary file 1 [file Table_1.DOCX]

Supplementary Material

**Supplementary Table 1.** The physicochemical properties of composting materials.

| Raw materials | MC (%) | TOC (%) | TKN (%) | C/N ratio | pH |
| --- | --- | --- | --- | --- | --- |
| DGW | 60.32±2.63 | 52.62±1.33 | 2.96±0.21 | 18.56±1.12 | 3.51±0.22 |
| Corncob | 8.2±0.24 | 53.27±2.01 | 0.53±0.15 | 99.55±1.18 | n.d. |

n.d. not determined.

^a^MC, Moisture content.

^b^TOC, Total organic carbon.

^c^TKN, Total Kjeldahl nitrogen.

**
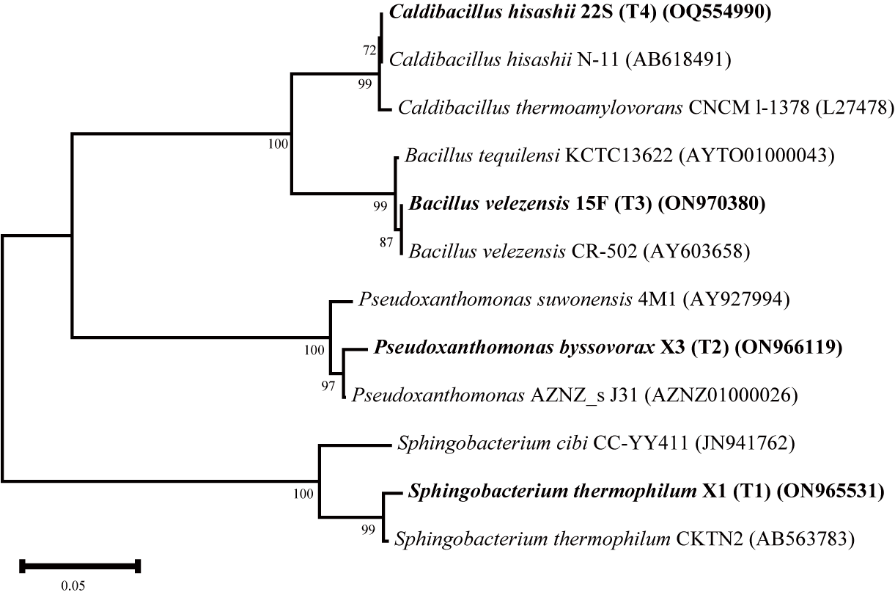
**

**Supplementary Fig 1.** Identification of the screened bacteria**.** The phylogenetic tree of X1, X3, 15F, and 22S was based on 16S rRNA gene sequencing using the neighbor-joining method.

**
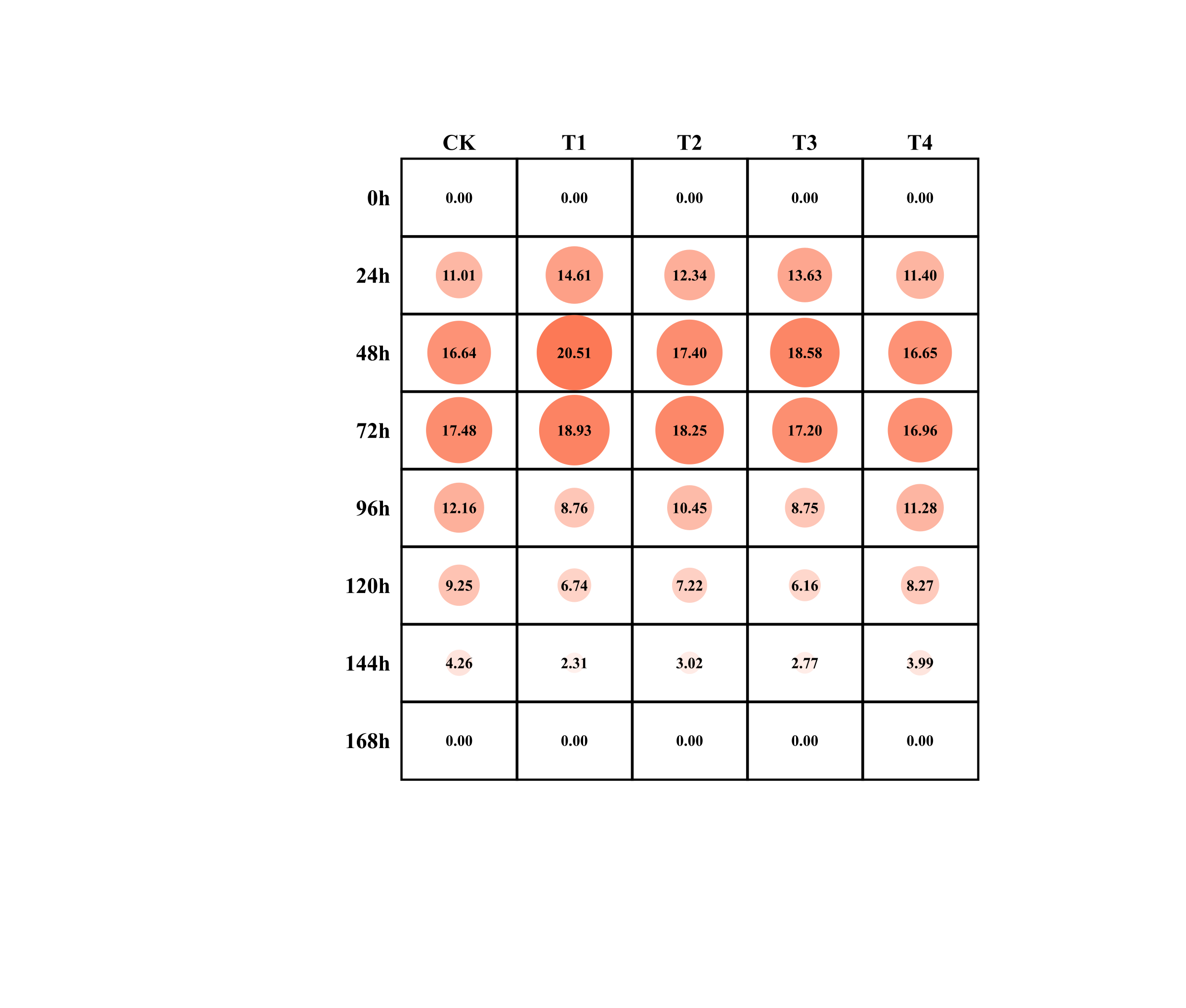
**

**Supplementary Fig 2** The ergosterol content (μg g^-1^) of *P. ostreatus* cultured in DGW substrates withdrawn every 24 h of composting.


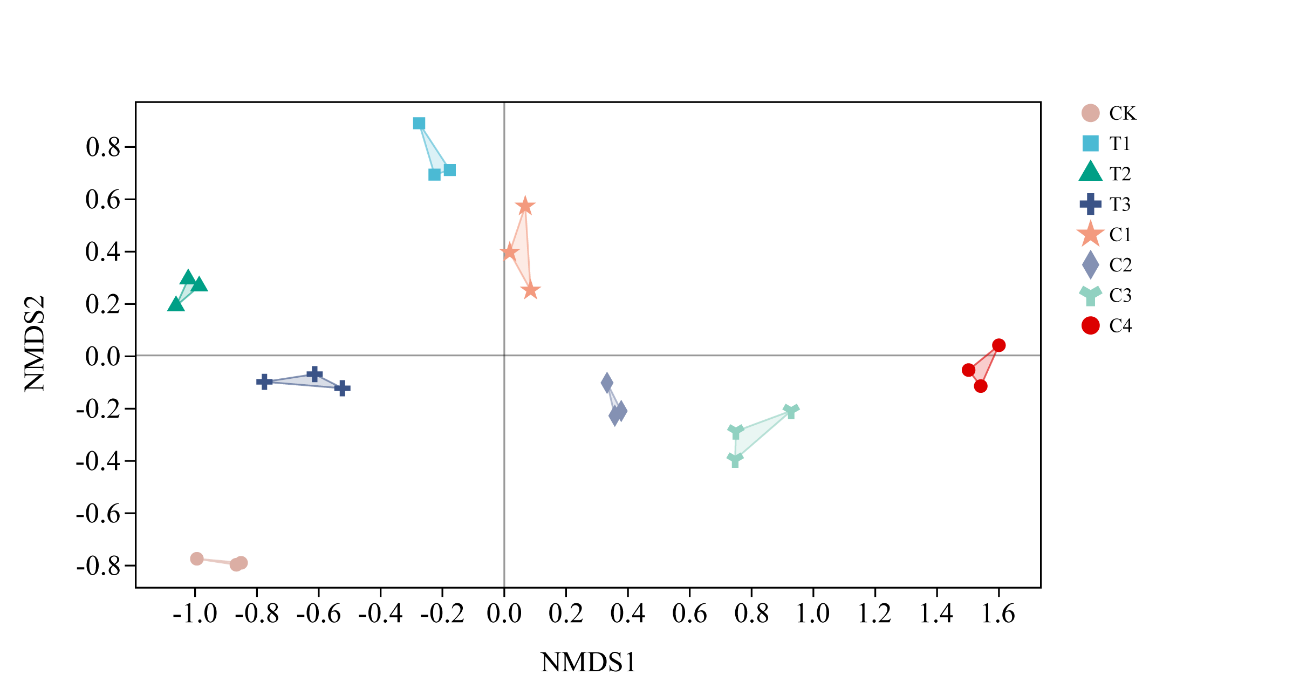


**Supplementary Fig 3** Non-metric multidimensional scaling (NMDS) analysis of the bacterial community composition in 12 h composted DGW samples with individual and combined inoculations.


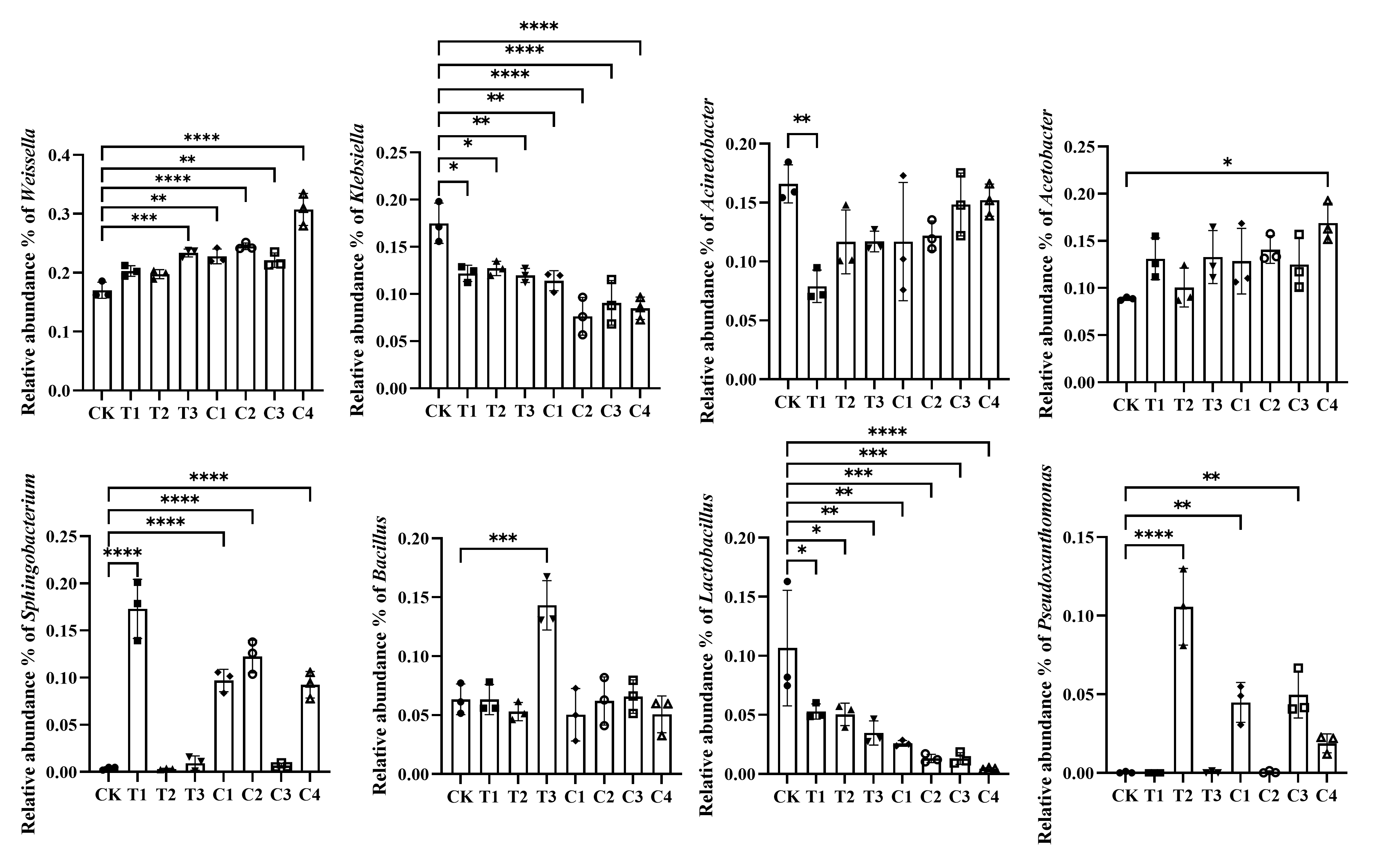


**Supplementary Fig 4** The relative abundance of top abundant genera in 12 h composted DGW samples with individual and combined inoculations.
